# Evaluation of Microleakage of RMGIC and Flowable Composite Immersed in Soft Drink and Fresh Fruit Juice: An *in vitro* Study

**DOI:** 10.5005/jp-journals-10005-1071

**Published:** 2010-09-15

**Authors:** Prabha Devi C Maganur, AR Prabhakar, Sugandhan S, Srinivas Namineni

**Affiliations:** 1 Ex-Postgraduate Student, Department of Pedodontics, Bapuji Dental College, Davangere, Karnataka, India; 2 Professor and Head, Department of Pedodontics, Bapuji Dental College, Davangere, Karnataka, India; 3 Professor, Department of Pedodontics, Bapuji Dental College, Davangere, Karnataka, India; 4 Professor, Department of Pedodontics, Bapuji Dental College, Davangere, Karnataka, India

**Keywords:** Erosion, Non-carious cervical tooth loss, Soft drink, Fresh fruit juice, Microleakage, Flowable composite, RMGIC.

## Abstract

**Aim :** The objective of the study was to evaluate and compare the effect of a soft drink and a fresh fruit juice on the microleakage of flowable composite and resin modified glass lonomer cement (RMGIC).

**Methods and materials :** 70 non-carious human premolars were collected and stored in saline until further use. Class-V cavities were prepared and restored with RMGIC on the buccal surface and flowable composite on the lingual surface for evaluating microleakage. The experimental groups (Group I and II ) comprised of 60 teeth, while the remaining 10 formed the control group (Group III―Water). The experimental groups were further divided into 2 groups (Group I―Cola drink and Group II―Fresh orange fruit juice) of 30 teeth each. Each group was then further divided into 3 subgroups (Short, Medium and Long-immersion) containing 10 teeth as shown in flow chart.

Immersion regime was followed according to Maupome G et al and microleakage was evaluated by using Rhodamine B dye and examined under stereomicroscope.

**Results :** Microleakage data obtained was statistically analyzed by Chi-square test. The teeth showed statistically significant microleakage as the immersion regime increased.

**Interpretation and Conclusion :** Low pH soft drink caused highly significant microleakage at the tooth and restorative material interface in medium and high immersion regimes signifying that the leakage pattern was directly proportional to the number of immersions. Thus, the study conclusively proves that the ‘sipping habit’ associated with commonly available low pH beverages is detrimental to the longevity of restorations.

## INTRODUCTION

The average daily requirement of water in human beings is 2-3 liters, of which, in developed countries, more than half comes from soft drinks. Commonly consumed soft drinks cause damage to the teeth due to their low pH and high titratable acidity leading to non-carious cervical tooth loss (NCTL). The sugars in these drinks are metabolized by plaque microorganisms to generate organic acids that bring about demineralization leading to dental caries.^1^

The commercial sale of soft drinks has increased by 56% over the last 10 years and now it is estimated that it will keep rising at about 2-3% a year.^1^ What with these soft drinks substituting water, their erosive effects on dental hard tissues and the subsequent NCTL pose a special challenge to any dentist for their restoration.

Over the last decade, the prevalence of dental erosion seems to have increased presumably due to an increase in the consumption of soft drinks and fruit juices.^2^ It has been recognized as an important cause of tooth structure loss not only in adults, but also in children.^3^

In NCTL, the coronal margins of cervical restorations are usually in enamel, while the cervical margins are in dentin and cementum. Dentin, unlike enamel, is a vital tissue and has higher organic content. A restorative system that bonds adequately to enamel and dentin is therefore desirable.^4^ A wide plethora of adhesive restorative materials is available for use these days, such as resin composites with their respective dentinal adhesive systems, RMGIC and Compomers. Therefore, the present study was conducted to evaluate the effects of cola drink (Coca Cola^®^), fresh fruit juice (Orange) on the microleakage of Filtek™ Flow (Flowable composite) and Vitremer™ Tri Cure (RMGIC).

## METHODOLOGY

**Fig. 1:**
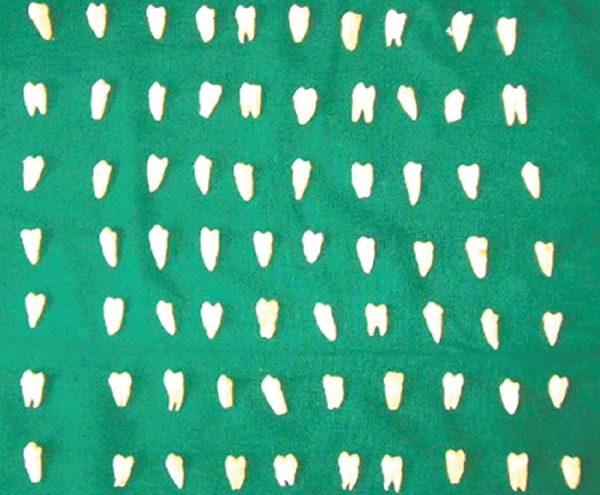
Microleakage test specimens

**Fig. 2:**
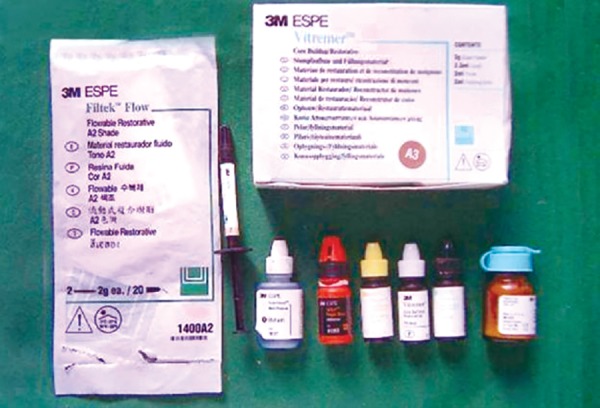
Restorative materials Filtek™ Flow and Vitremer™

**Fig. 3:**
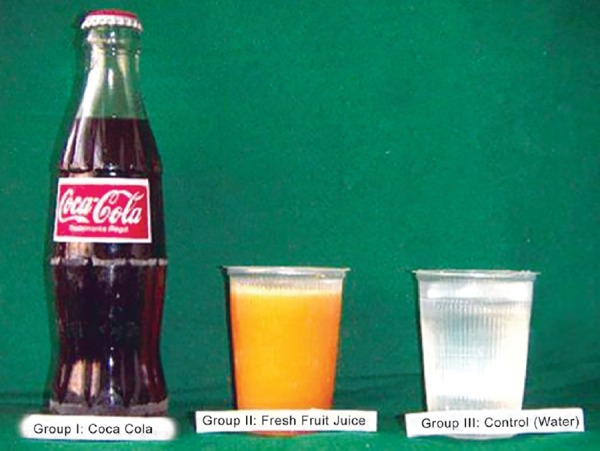
Experimental drinks: Group I: Cola drink (Coca Cola^®^), Group II: Fresh fruit juice (Orange), Group III: Water (Control)

Seventy human premolars with no signs of caries or developmental defects extracted for orthodontic treatment purpose were used (Fig. 1). Two restorative materials namely Vitremer™ Tri Cure and Filtek™ Flow (Fig. 2) and soft drink (Coca Cola^®^) and fresh fruit orange and water as control (Fig. 3) were used for the study.

Standardized Class-V cavities (3 mm in length, 2 mm in width and 1.5 mm in depth) were prepared on the buccal and lingual surfaces of the teeth, 1 mm above the CEJ. The cavity preparation was standardized using a William’s graduated periodontal probe.^5^

The cavities on the buccal surface were restored with Vitremer™ Tri Cure restorative material. Vitremer™ Primer (3M Dental Products) was applied on the cavity walls for 20 seconds; gently air dried and light cured for 30 seconds. Vitremer™ Tri Cure (3M Dental Products) was mixed according to the manufacturer’s instructions and placed in the cavity. Immediately after the restorative material was placed, a Mylar strip was adapted over the restoration and cured with a visible light source for 40 seconds. The matrix was removed and the restorations were finished with a fluted carbide bur and polished with a wet abrasive disk (Sof-Lex™, 3M Company). After finishing, the restorations were gently air-dried and a layer of unfilled resin (Vitremer™ gloss 3M Company) was applied and light cured.^6^

The cavities on the lingual surface were restored with Filtek™ Flow material. After etching the enamel and dentin with 34.5% phosphoric acid for 15 seconds, the cavities were thoroughly rinsed with water for 15 seconds. They were then air dried gently for 5 seconds to avoid complete desiccation. Two consecutive coats of Single Bond (3M Dental Products) were applied to the whole cavity surface followed by gentle air-drying to remove excess solvent and light cured for 10 seconds. The cavities were filled with flowable resin composite; Filtek™ Flow (3M Dental Products) and cured for 20 seconds.^5^

The restored teeth were stored in water at room temperature for 1 week. During this period, the teeth were subjected to 200 thermocycles between 5°C and 55°C water baths. Dwell time was 1 minute with 10 seconds transit between baths. Then the samples were subjected to the various immersion regimens.^7^

**Fig. 4:**
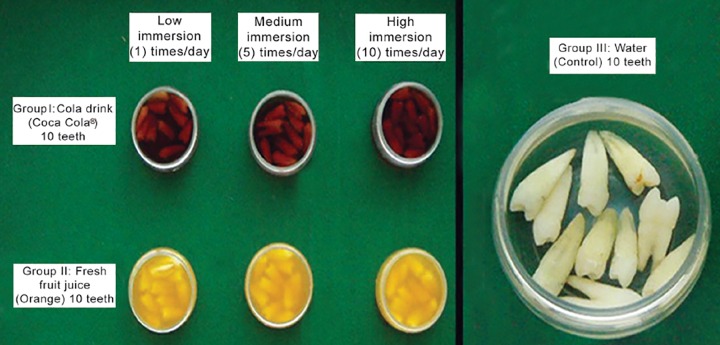
Immersion regime employed to evaluate the microleakage of the specimen

Out of the 70 prepared tooth samples, 60 were equally divided into two groups of 30 each. Each group was further divided into 3 subgroups as mentioned below. The remaining 10 prepared samples were used as control and immersed in water and named as Group III (Fig. 4).

**Table:** 

*Groups*		*Low* *immersion* *(1 time/day)*		*Medium* *immersion* *(5 times/day)*		*High* *immersion* *(10 times/ day)*	
Group I Cola drink (Coca Cola^®^)		10		10		10	
Group II fresh fruit juice (Orange)		10		10		10	

Immersion regimes were meticulously carried out for 8 days; each immersion lasting for five minutes. For medium immersion and high immersion regimes, the regime was evenly distributed over a 12-hour period. Before and after each immersion, the restorations were copiously rinsed in 0.1 M phosphate buffered saline (PBS pH 7.2). When not exposed to the immersion regime, they were stored in deionized water at room temperature.

**Fig. 5:**
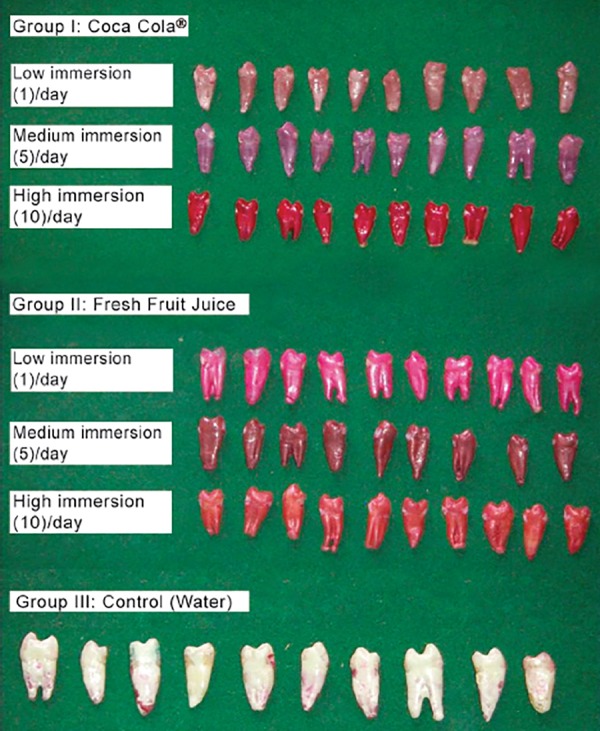
Specimens following nail varnish application

**Fig. 6:**
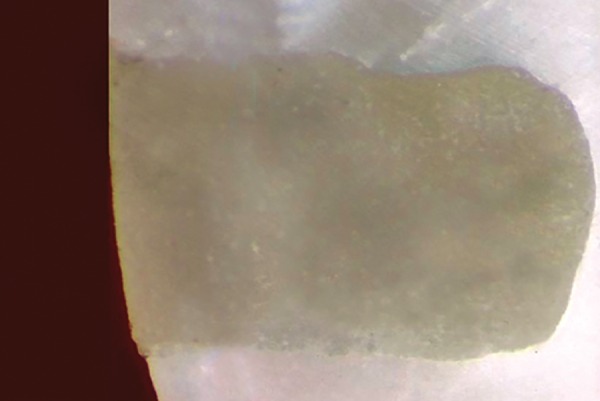
Score 0: No dye penetration

**Fig. 7:**
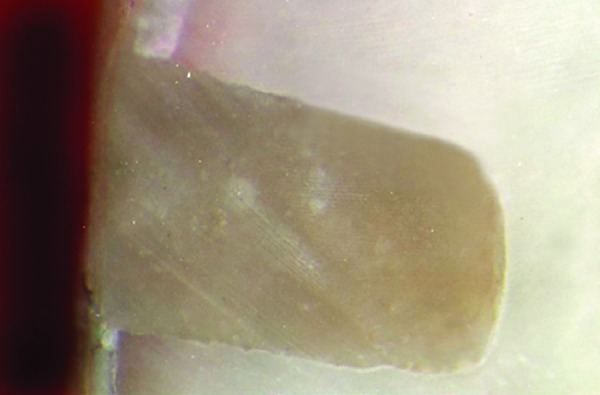
Score 1: Dye penetration along occlusal wall but less than // way to axial wall

**Fig. 8:**
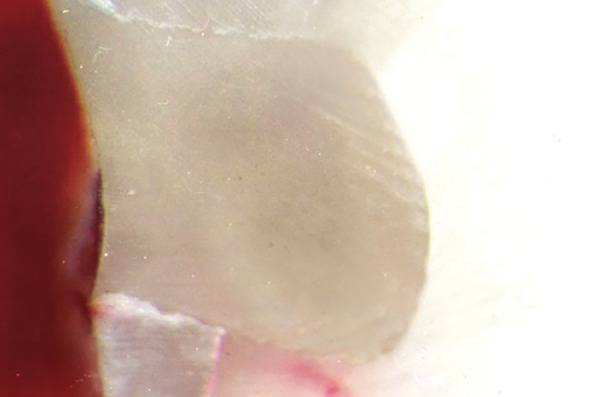
Score 2: Dye penetration along occlusal wall but more than 1 way to axial wall

**Fig. 9:**
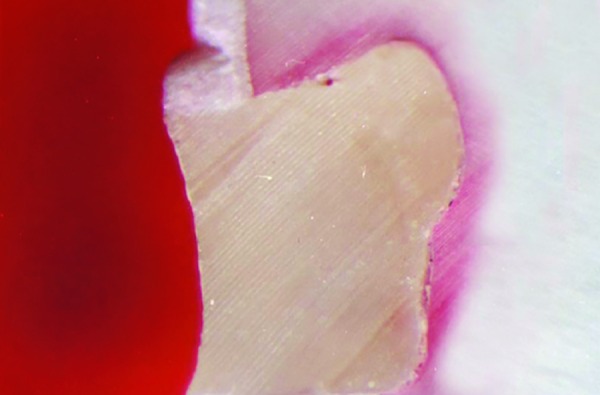
Score 3: Dye penetration along occlusal wall, up to and along axial wall

At the end of the test period, the apices of the teeth were sealed with sticky wax, and all tooth surfaces except a 1 mm wide zone around the margins of the restoration (bucally and lingually) were painted with nail varnish (Fig. 5). To minimize dehydration of the restorations, the teeth were replaced in deionized water as soon as the nail varnish dried. The teeth were then immersed in 1% Rhodamine B solution (pH- 7.2) for 24 hours at 37°C and rinsed, dried, and finally invested in clear resin.^8^ Each tooth was sectioned bucco- lingually through the center of the restoration with help of a low speed water cooled diamond disk. The specimens obtained were examined under a stereomicroscope to evaluate the microleakage. Dye penetration was graded based on the extent of penetration along the occlusal wall of the restoration using the criteria recommended by Michal Staininec and Mark Holtz (1988).^9^ Scores (Figs 6 to 9)

**Table:** 

Score 0		No dye penetration	
Score 1		Dye penetration along occlusal wall but less than ½ way to axial wall	
Score 2		Dye penetration along occlusal wall but more than ½ way to axial wall	
Score 3		Dye penetration along occlusal wall, up to and along axial wall	

In order to avoid bias, scoring of the samples was done by a single blinded investigator on two different occasions and the average scores obtained were tabulated and statistically analyzed.

## RESULTS

**Filtek™ Flow (Table 1)**

### Group I: Cola Drink (Coca Cola^®^) (Table 1 and Graph 1)

In low immersion regime, 4 specimens (40%) scored 0 and 6 specimens (60%) scored 1. In the medium immersion regime, all the 10 (100%) specimens scored 2. In high immersion regime, 2 (20%) specimens scored 2 and 8 (80%) specimens scored 3.

**Table 1:** **Table 1:** Descriptive statistics on the microleakage pattern of Filtek™ Flow following immersion for varying periods of time in Cola drink (Coca Cola^®^), Fresh fruit juice (Orange) and Water (Control)

*Groups*	*Microleakage* * scores*	*Low* * immersion*		*Medium* * immersion*		*High immersion*		*Difference between Immersion* * regime*		
		*No*	*Mean* * median*	*No*	*Mean* * median*	*No*	*Mean* * median*	*Low vs* * medium*	*Low vs* * high*	*Medium vs* * high*
*Group I - * *Cola drink* * (Coca* * Cola^®^)*	*0*	*4*	*0.6* *1.0*	*-*	*2.0* *2.0*	*-*	*2.8* *3.0*	*P< 0.001* *HS*	*P< 0.001* *HS*	*P< 0.001* *HS*
	*1*	*6*		*-*		*-*				
	*2*	*-*		*10*		*2*				
	*3*	*-*				*8*				
*Group II - * *Fresh fruit* * juice* * (Orange)*	*0*	*10*	*0.0* *0.0*	*7*	*0.3* *0.0*	*1*	*0.9* *1.0*	*P> 0.005* *NS*	*P< 0.001* *HS*	*P< 0.001* *S*
	*1*	*-*		*3*		*9*				
	*2*	*-*		*-*		*-*				
	*3*	*-*		*-*						
*Group III - * *Water* *(Control)*	*No immersion regime was followed*									
	*0*	*10*	*0.0* *0.0*								
	*1*	*-*									
	*2*	*-*									
	*3*	*-*									
*Differences* *between* *groups*	*Group I - II*	*P<0.01* *s*		*p < 0.001 HS*		*P< 0.001 HS*				
	*Group I - III*	*P<0.01* *s*		*p < 0.001 HS*		*P< 0.001 HS*				
	*Group II - III*	*p > 0.05 NS*		*p>0.05 NS*		*P< 0.001 HS*				

Chi-square test/Fisher’s exact test

p < 0.05, p < 0.01: Significant (S), p < 0.001: Highly significant (HS), p > 0.05: Not significant (NS)

Intragroup Comparison

When specimens were compared between three immersion regimes, all the specimens scored higher microleakage as the number of immersion intervals increased indicating that higher the time of immersion, more is the leakage. The results were statistically highly significant (p < 0.001).

### Group II: Fresh fruit juice (Orange) (Table 1 and Graph I)

All the 10 (100 %) specimens scored 0 in the low immersion regime. In the medium immersion regime, 7 (70%) specimens scored 0 and 3 (30%) specimens scored 1. In high immersion regime, only 1(10%) specimen showed a score of 0 and 9 (90%) specimens indicated score 1.

Intragroup Comparison

There was no statistically significant leakage in the specimens when compared between the low and medium immersion regimes, but statistically significant leakage (p < 0.01) when compared between medium and high immersion regime and statistically highly significant leakage (p < 0.001) when compared between low and high immersion regimes.

### Group III: Water (Control) (Table 1 and Graph I)

The specimens of this group were not subjected to any immersion regime. All the 10 (100%) specimens scored scores 0.

Intergroup Comparison of Different Immersion Regimes

Amongst all groups, the specimens showed statistically significant leakage (p < 0.01) in low immersion regime with Group-I showing more leakage compared to Group-II and Group-III. The specimens scored statistically highly significant leakage (p < 0.001) in medium and high immersion regimes with Group-I showing more leakage compared with Group-II and Group-III.

Comparing Group-II with Group-III in low and medium immersion regimes, the specimens showed statistically no significant (p > 0.05) leakage, but in high immersion regime, the Group-II specimens showed statistically highly significant leakage (p < 0.001).

**Table 2:** **Table 2:** Descriptive statistics on the microleakage pattern of Vitremer™ following immersion for varying periods of time in Cola drink (Coca Cola^®^), Fresh fruit juice (Orange) and Water (Control)

*Groups*	*Microleakage* * scores*	*Low* * immersion*		*Medium* * immersion*		*High immersion*		*Difference between Immersion* * regime*		
		*No*	*Mean* * median*	*No*	*Mean* * median*	*No*	*Mean* * median*	*Low vs* * medium*	*Low vs* * high*	*Medium vs* * high*
*Group I - * *Cola drink* * (Coca Cola^®^)*	*0*	*7*	*0.3* *0.0*	*-*	*1.1* *1.0*	*-*	*2.2* *2.0*	*P < 0.001* *S*	*P < 0.001* *S*	*P < 0.001* *S*
	*1*	*3*		*9*		*-*				
	*2*	*-*		*1*		*8*				
	*3*	*-*		*-*		*2*				
*Group II - * *Fresh fruit* * juice* * (Orange)*	*0*	*9*	*0.1* *0.0*	*8*	*0.2* *0.0*	*9*	*0.1* *0.0*	*P > 0.005* *NS*	*P > 0.005* *NS*	*P > 0.005* *NS*
	*1*	*1*		*2*		*1*				
	*2*	*-*		*-*		*-*				
	*3*	*-*		*-*		*-*				
*Group III - * *Water* *(Control)*	*No immersion regime was followed*									
	*0*	*10*	*0.0* *0.0*								
	*1*	*-*									
	*2*	*-*									
	*3*	*-*									
*Differences* *between* *groups*	*Group I - II*	*P > 0.05 S*		*P < 0.01 S*		*P < 0.01 S*				
	*Group I - III*	*P > 0.05 HS*		*P < 0.01 HS*		*P < 0.01 HS*				
	*Group II - III*	*P > 0.05 NS*		*P > 0.05 NS*		*P > 0.05 HS*				

Chi-square test / Fisher’s exact test

p < 0.05, p < 0.01: Significant (S), p < 0.001: Highly significant (HS), p > 0.05: Not significant (NS)

## VITREMER™ (TABLE 2 AND GRAPH 2)

### Group I: Cola Drink (Coca Cola^®^) (Table 2 and Graph 2)

In the low immersion regime, 7 (70%) specimens scored 0 and 3 (30%) scored 1. In medium immersion regime 9 (90%) specimens indicated score of 1 and only 1 (10%) specimen scored 2. In the high immersion regime 8 (80%) specimens scored 2 and only 2 specimens (20%) scored 3.

Intragroup Comparison

The leakage pattern tends to increase in proportion with the number of times of immersion. The results scored were statistically significant (p < 0.01) between low, medium and high immersion regimes.

### Group II: Fresh Fruit Juice (Orange) (Table 2 Graph II)

In the low immersion regime 9 (90%) specimens scored 0 and only 1 (10%) scored 1. In the medium immersion regime 8 (80%) specimens scored 0 and only 2 (20%) specimens scored 1. In high immersion regime 9 (90%) specimens scored 0 and only 1 specimen (10%) scored 1.

Intragroup Comparison

There was no significant leakage pattern between low, medium and high immersion regimes. The results were not statistically significant (p > 0.05).

### Group III: Water (Control) (Table 2 and Graph II)

The specimens of this group were not subjected to any immersion regime. All the 10 (100%) specimens scored 0.

Intergroup Comparison of Different Immersion Regimes

The Group I specimens when compared to Group II and Group III, Group II specimens compared to Group III did not show any statistically significant leakage (p > 0.05) in low immersion regime. The Group II specimens compared to Group III scored no statistically significant leakage scores (p > 0.05) in medium and high immersion regimes. The Group I specimens showed statistically significant leakage (p < 0.01) when compared with Group II and Group III in both medium and high immersion regimes.

## DISCUSSION

Dental erosion is defined as an irreversible loss of dental hard tissue by a chemical process without the involvement of microorganisms and is due to either extrinsic or intrinsic sources.^10^ Enamel, inspite of being the hardest tissue, has been reported to suffer from the devastating effects of soft drinks.^11^

Dietary erosion may result from food or drinks containing a variety of acidic ingredients. Frequent consumption of these easily and widely available beverages showed erosion of the enamel in both *in vitro* and *in vivo* studies.^12-17^

Most carbonated beverages and sport drinks have a pH below 3.5 and experiments have shown that enamel dissolution occurs below pH4.^18^ Dental caries along with dental erosion being a one-way irreversible destruction of the tooth, replacement of the lost tissue would be the only available option.

Phosphoric acid is a common constituent of most of the soft drinks.^11,18,19^ The acid content of the cola soft drink, which is added to give a peculiar tangy taste and has a preservative property, is known to play a well-established role in the erosive process. Substances in cola soft drinks absolutely affect the integrity of the enamel surface.^1^

In our study, we used two commonly consumed beverages, i.e carbonated drink as well as fresh fruit juice. This was done because the acidogenic potential of the commonly consumed fresh fruit juice (Orange pH 3.98)^13^ and carbonated drink (Coca Cola pH < 3.5) was less than 4.^18^

In the oral environment, both dissolution of elements and erosion of the nonsoluble components of the restorative materials occur. Numerous factors like low pH, acidic foods, ionic composition, ionic strength of saliva and enzymatic attack are important parameters, which may influence the quality and the quantity of the substances released from a restorative material; as well as its physical and mechanical characteristics.^20^

Considering these concepts, this current study was carried out to evaluate the microleakage of Filtek™ Flow and Vitremer™ after immersing for varying periods of time in a cola drink (Coca Cola^®^) and fresh fruit juice (Orange).

In this study, Class V cavities were prepared on the buccal and the lingual surfaces of extracted human premolars 1 mm above the CEJ^5^ this is to reduce the microleakage because cavities place 1 mm below the CEJ have shown significantly more leakage due to the inadequate bonding of the restorative material to the tooth structure.^21,22^

The immersion regime followed in our study was about 5 minutes contralateral to those studies that had employed extremely long immersion regime ranging from 15 minutes to 72 hours.^11,15,16,23,24^ This was done to ascertain more realistic consumption pattern replicated under experimental conditions, which would be helpful in determining the actual impact of soft drinks by resembling real time exposure.^8^

Before and after each immersion in Cola drink (Coca Cola^®^) and fresh fruit juice (orange), the specimens and pellets were copiously rinsed in 0.1 M phosphate buffered saline (PBS, pH 7.2)^8,25^ to buffer the effect of Cola drink (Coca Cola^®^) and fresh fruit juice (orange) after the prescribed exposure time. This was done to return pH to a neutral level once the exposure was over and to avoid prolonged insult to the materials while they were stored in the deionized water.

The microleakage pattern of Filtek™ Flow in the Cola drink group increased as the number of immersion regimes increased. Filtek™ flow in the fresh fruit juice (orange) group showed statistically significant leakage between medium and high immersion regimes.

On comparing the microleakage scores of Filtek™ flow when exposed to the Cola group, fresh fruit juice (orange) group and water (control) under the low, medium and high immersion regimes, it was evident that the microleakage showed an increasing trend in the Cola group as compared to the fresh fruit juice and control groups. Cola beverages contain phosphoric acid as the main acid which has pH of 2.57 ^18,26,27^ as compared to the higher pH of fresh orange juice (pH- 3.98)^28^ and neutral pH of water. Therefore, the acidic nature of the cola drink would have affected the integrity of the restoration/enamel and increased the microleakage.

Similarly, the microleakage pattern of Vitremer™ in the Cola Drink Group increased as the number of immersion regimes increased which could be attributed to reasons cited above. There were no significant differences in the microleakage scores between the low, medium and high immersion regimes of Vitremer™ in the fresh fruit juice group. Studies involving the evaluation of fresh fruit juice have reported that though orange juice is rich in citric acid, but it has a pH of 3.98.^28^ This pH is just about the critical pH drop below 4 needed to cause enamel erosion.^18^ Vitremer is a light cured RMGIC, which was developed to improve the handling and working characteristics of the original glass ionomer formulation. Though these materials are known to set by a dual cure mechanism, like glass ionomer but they are believed to release fluoride. This could be the possible reason that there was no significant leakage seen around Vitremer in the fresh fruit juice (Orange) group.

**Graph 1:**
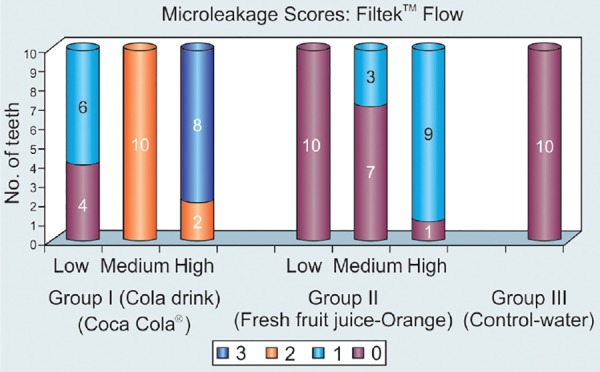
Descriptive statistics on the microleakage pattern of Filtek™ Flow following immersion for varying periods of time in Cola drink (Coca Cola^®^), Fresh fruit juice (Orange) and Water (Control)

**Graph 2:**
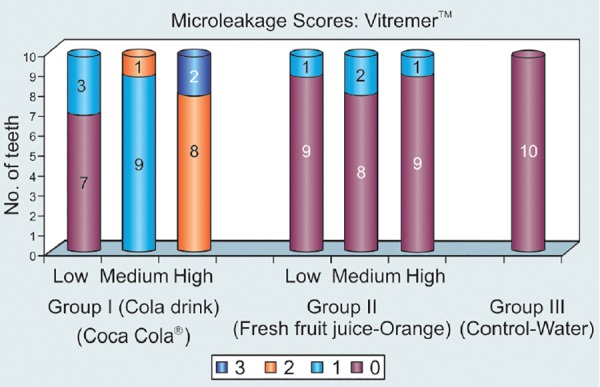
Descriptive statistics on the microleakage pattern of Vitremer™ following immersion for varying periods of time in Cola drink (Coca Cola^®^), Fresh fruit juice (Orange) and Water (Control)

The Vitremer™ restoration specimens showed a trend of increasing microleakage as the number of immersions increased especially in the Cola group^23^ from their study inferred that as the period of exposure increased, so did the severity and depth of erosive lesions.^23^ The pH of the acidic drinks also played a major role, as it might be true that higher microleakage scores were due to much of the restorative materials being removed along with the enamel.^11^

However, it is inappropriate to extrapolate the findings of our study to the conditions existing *in vivo* in humans. In the oral cavity, any drink or foodstuff will be instantaneously mixed with saliva with a subsequent rise in its pH. After consuming a low pH drink, the pH on the tongue stays low only for a short duration. In addition, acidic drinks have also been shown to stimulate salivary secretion, which in turn facilitates the buffering systems.^18^

However, we recommend further studies combining both qualitative and quantitative evaluations, which will indicate more precisely the effects of fruit beverages on the clinical integrity of restorative materials in the oral environment.
